# Inferring True COVID19 Infection Rates From Deaths

**DOI:** 10.3389/fdata.2020.565589

**Published:** 2020-10-15

**Authors:** Ian McCulloh, Kevin Kiernan, Trevor Kent

**Affiliations:** ^1^Discovery Lab, Applied Intelligence, Accenture, Washington, DC, United States; ^2^Whiting School of Engineering, Johns Hopkins University, Laurel, MD, United States

**Keywords:** COVID19, Monte - carlo method, epidemiology, SARS - CoV-2, social distancing

## Abstract

The novel coronavirus, SARS-CoV-2, commonly known as COVID19 has become a global pandemic in early 2020. The world has mounted a global social distancing intervention on a scale thought unimaginable prior to this outbreak; however, the economic impact and sustainability limits of this policy create significant challenges for government leaders around the world. Understanding the future spread and growth of COVID19 is further complicated by data quality issues due to high numbers of asymptomatic patients who may transmit the disease yet show no symptoms; lack of testing resources; failure of recovered patients to be counted; delays in reporting hospitalizations and deaths; and the co-morbidity of other life-threatening illnesses. We propose a Monte Carlo method for inferring true case counts from observed deaths using clinical estimates of Infection Fatality Ratios and Time to Death. Findings indicate that current COVID19 confirmed positive counts represent a small fraction of actual cases, and that even relatively effective surveillance regimes fail to identify all infectious individuals. We further demonstrate that the miscount also distorts officials' ability to discern the peak of an epidemic, confounding efforts to assess the efficacy of various interventions.

## Introduction

Over 26 million people have been confirmed to be infected by COVID19 with over 864,000 dead as of 1 September, 2020, according to Johns Hopkins University's Coronavirus Research Center (Dong et al., 2020). National and local governments rely on forecasts and models to make decisions about interventions to slow the spread of the disease and prevent deaths. There are two broad classes of models used for this purpose, empirical, and mechanistic models. Empirical models fit a response surface to a dependent variable or multiple-response objective function using multiple input variables. The Institute for Health Metrics and Evaluation COVID19 model is probably the most popular and most accurate empirical model for COVID19 (Institute for Health Metrics Evaluation (IHME), 2020). While empirical models can provide accurate forecasts in the short term, there are several drawbacks to this approach. Empirical models can be overfit, where input variables are highly correlated or spurious. These models are also based on current observed conditions and may not be accurate past observed data or if the underlying conditions change. Mechanistic models are mathematical descriptions of a phenomenon or process based on an understanding or theory of how a system behaves, in which the structure of the model restricts the shape of the potential response surface. They are also easily parameterized in terms of behaviors that affect the reproductive rate of the disease, therefore lending themselves to the modeling of public health scenarios. However, they are generally very sensitive to variations in initial conditions, making the accurate estimation of those conditions a critical task in building useful models.

One of the most nettlesome challenges for researchers seeking to model the COVID19 epidemic is the poor quality of data on the number of COVID19 infections. This is likely due to a combination of factors, such as the novel nature of the disease, the lack of adequate testing, and the prevalence of asymptomatic cases. For example, we use publicly available data from the Johns Hopkins University Coronavirus Resource Center (Dong et al., 2020). As of 1 September, their raw data suggests a fatality rate of over 3.3%, which is inconsistent with numerous clinical studies on the disease. Several studies have shown that there is a large number of people who are infected with COVID19 yet show no symptoms and many not even be aware that they have been infected (Day, 2020; Hu et al., 2020; Kimball, 2020; Lavezzo et al., 2020; Mizumoto et al., 2020). In addition, many of those infected may not seek medical treatment either because symptoms are not severe or fear of exposing themselves to COVID19 infected patients. These factors make it very difficult for epidemiologists to understand accurate initial conditions needed for many epidemic models of disease propagation.

Of all the data available on COVID19 infections, we posit that the number of deaths per day is a more accurate source for estimating the number of infections. We assume that when a person dies, their death is recorded, and that the cause is attributed to COVID19 with reasonable accuracy. “Reasonable accuracy” in this case may be within +/50%, as most states are simultaneously recording increases in all-cause mortality that exceed recorded COVID19 deaths by a wide margin. There is speculation that some patients with pre-existing conditions, exacerbated by COVID19 are included, or that some patients with COVID19 have their cause of death attributed to non-COVID issues, such as pneumonia or COPD (Li et al., 2020). Nevertheless, we assert that the death counts are much more accurate than confirmed case counts, which fail to reflect the majority of COVID cases.

**Figure 1:**
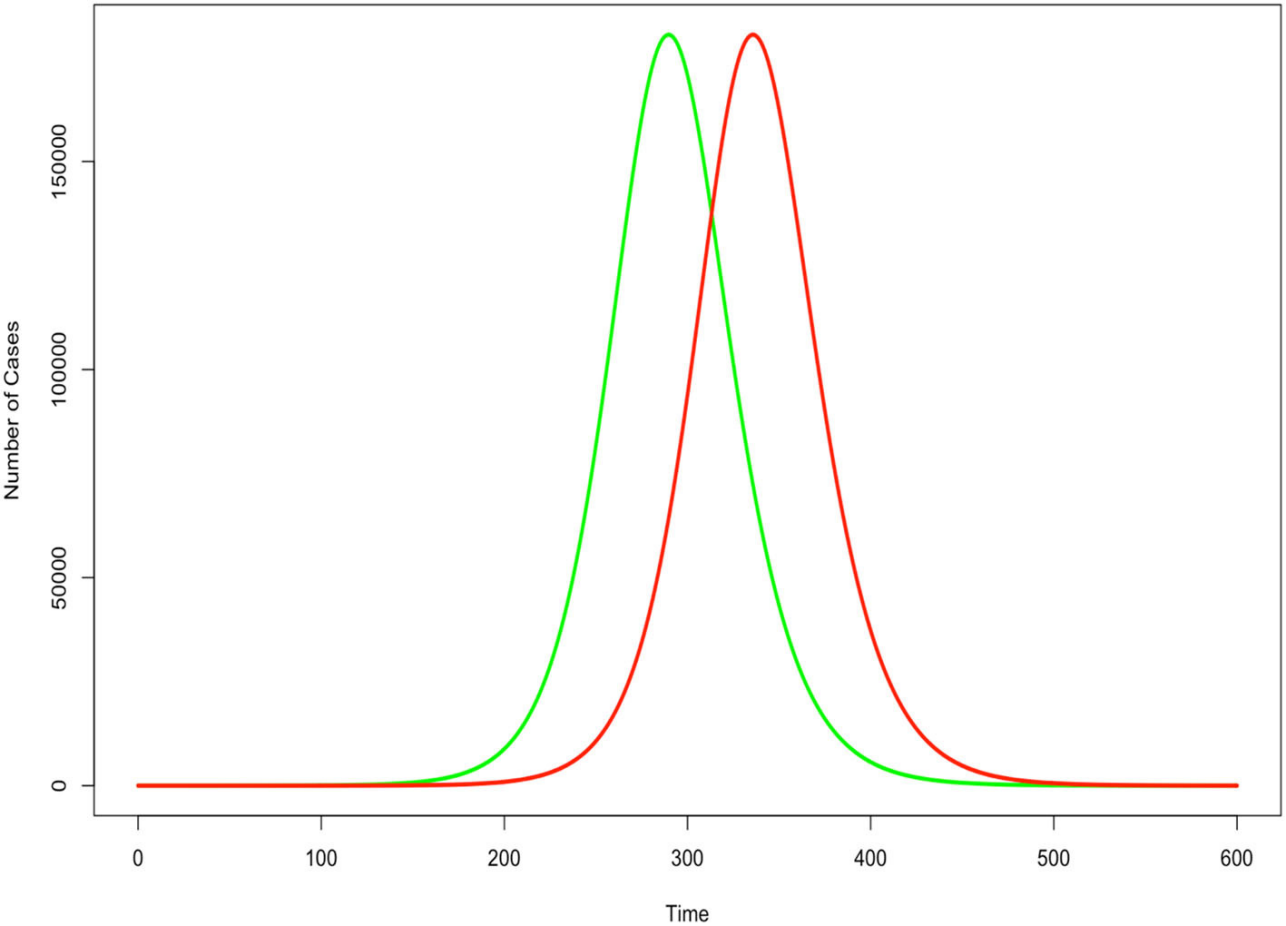
Impact of underestimated case count in an SIR epidemic model. Green indicates correct count for initial condition. Red indicates undercount. All other factors held constant.

An accurate estimate of those infected is important for policy decisions. Perhaps the most important reason is that the assumption that the fraction of the population infected directly impacts the estimates of disease duration. Figure 1 displays a notional susceptible-infected-recovered (SIR) model assuming a transmission rate of 0.5 and recovery period of 5 days. The two lines differ only in the initial condition of the fraction of the population infected, where the green line estimates 10 times as many people are infected as the red line. The infection cycle peaks and expires 46 days sooner when we assume 10 times more people are initially infected. Therefore, it is very important for policy makers and epidemiologists to have a more accurate understanding of true infection counts in order to effectively model the epidemic cycle and derive more realistic expectations of when the disease has reached its peak and subsided.

This paper is organized as follows. The method section proposes a novel method to estimate the true number of infected cases using reported death data, parameters derived from clinical literature, and the application of Monte Carlo simulation. The results section provides results for New York State for expository purposes. Similar findings exist for all U.S. states. The discussion section provides insights and considerations for policy makers. This method provides an approach to correct significant under-reporting in confirmed COVID19 cases, which has major implications for policy decisions affecting the lifting of social distancing measures and reopening major local and global economies.

## Method

The proposed approach is conceptually simple: estimate the number of cases each death represents using infection fatality ratios (IFR) derived from clinical literature, then estimate the delay between infection and death using time-to-death (TTD) distributions derived from the same sources. Uncertainty is estimated by performing Monte Carlo (MC) draws from the two distributions and identifying the median and 95% confidence interval. Note that the uncertainty estimates derived from this process reflect the uncertainty in the distribution parameters, not the likely undercount of COVID19 deaths.

We obtain estimates of IFR, which analyzed the Wuhan and *Diamond Princess* outbreaks, respectively (Russell et al., 2020; Verity et al., 2020). Verity et al. (2020) estimates an IFR of 0.66% with a 95% confidence interval [0.389, 1.33]. The same paper estimated the mean TTD as 17.8 days. Russell et al. (2020) estimated an IFR of 1.3% on the 95% confidence interval [0.38, 3.6].

COVID-19's documented tendency to produce highly infectious but nonetheless asymptomatic individuals poses a unique challenge for public health officials attempting to perform disease surveillance. A recent community testing program in the Italian municipality of Vo, Italy revealed that 42.5% of infected individuals exhibited no symptoms (Lavezzo et al., 2020). We use the 42.5% asymptomatic figure to estimate the number of individuals who are likely to be detected via testing of people exhibiting symptoms. Comparison between the uncertainty range of symptomatic cases and observed case counts provides a measure of how well public health officials are able to detect the progression of a COVID outbreak. The symptomatic fraction is presumably the most suitable data series to compare with case count data, as people are far more likely to interact with the medical system when they feel sick. The same study also found that the viral load of SARS-Cov-2 was not significantly different for the asymptomatic and symptomatic populations (Lavezzo et al., 2020). Researchers have attempted to model the asymptomatic population as a separate compartment (Arenas et al., 2020). However, new evidence suggests that the viral load of the asymptomatic population isn't significantly different from the symptomatic population (Lavezzo et al., 2020; Zou et al., 2020). This validates the utility of our method in that a researcher seeking to model the future evolution of the disease with a traditional mechanistic model can do so with a single infectious compartment and use the prevalence data stemming from this technique for initial conditions.

Our analysis accounts for the uncertainty in the literature-estimated parameters by treating them as distributions to sample from. For each MC draw, we assign a value of IFR by sampling from the log-normal distribution

$$\begin{array}{l} IFR=\ln N\left(1,\mu ,\sigma \right) \end{array}$$

where μ and σ are the log of the mean and standard deviation of the distribution.

We use the IFR to assign a number of imputed infections to each death. We estimated the number of daily infections *I*_*d*_ as,

$$\begin{array}{l} I_{d}=1/IFR^{*}D_{d} \end{array}$$

where *IFR* is a percentage and *D*_*d*_ are the daily number of deaths, respectively.

Having estimated the *number* of infections represented by each death, we now model the time it took for each person to die, and thereby simulate the likely date of infection for each case:

$$\begin{array}{l} lags_{d}=\ln N\left(I_{d},\delta_{\mu },\delta_{\sigma }\right) \end{array}$$

*lags*_*d*_ is a vector containing the number of days in the past each infection represented by *I*_*d*_ began, and δ_μ_ and δ_σ_ are the log of the mean and standard deviation of time to death, respectively.

For each MC run, we convert the lags into a list of dates, then count the number of cases per date. We then perform 500 MC runs, and summarize the results by identifying the median, 2.5th percentile, and 97.5th percentile values of new infections each day.

This method is validated by a recent serology survey from New York state. The survey developed a baseline infection rate by testing 15,000 people at grocery stores and community centers across the state over the last 2 weeks of April 2020 (New York Press Office, 2020). Of those tested, 11.5% of women tested positive, compared to 13.1% for men, or a 12.3% total population infection rate.

This paper does not consider age or socioeconomic strata as inputs to our analysis, though it would be conceptually straightforward to do so, provided sufficiently granular data. A researcher possessing IFR estimates, case counts, and death counts for each proposed stratum could repeat our analysis and aggregate the results for any given region of interest. Similarly, it may be possible to refine our results by examining discrepancies in the administration of death records and attempting to correct them. While these are potentially worthwhile avenues for further research, they are beyond the scope of this paper.

## Results

**Figure 2:**
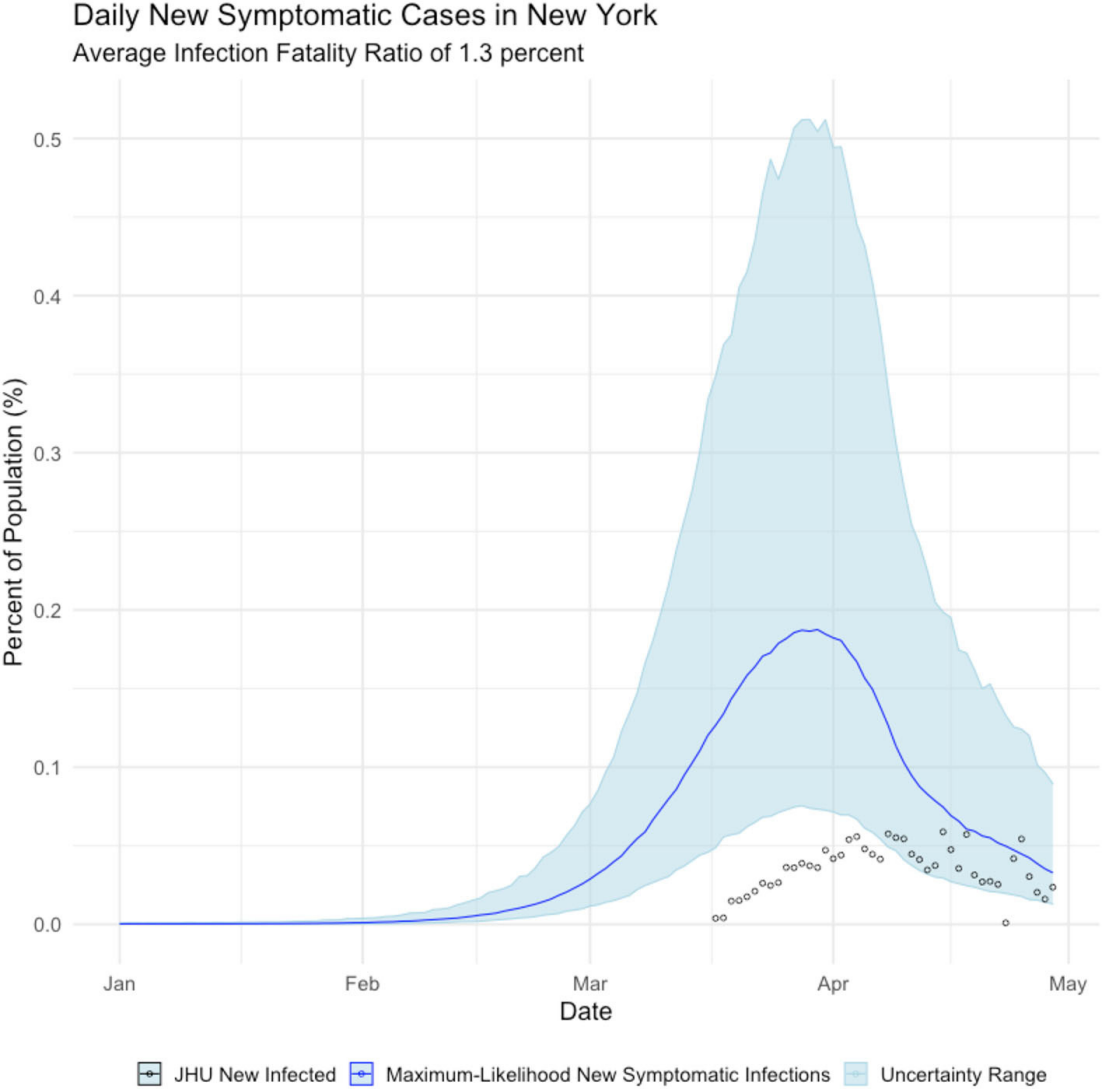
Daily Count of New Infections, New York state. Blue line is the maximum-likelihood estimate of new symptomatic infections. Black circles are observed cases counts.

We estimated infections for all 50 states and Washington, D.C. For expository purpose, we present the results for New York state using IFR estimates from the *Diamond Princess* of 1.3%. In Figure 2, we present our estimate of daily new symptomatic cases overlaid with observed case counts. Based on this comparison, we assert that COVID cases in New York are systematically undercounted, as shown below.

**Figure 3:**
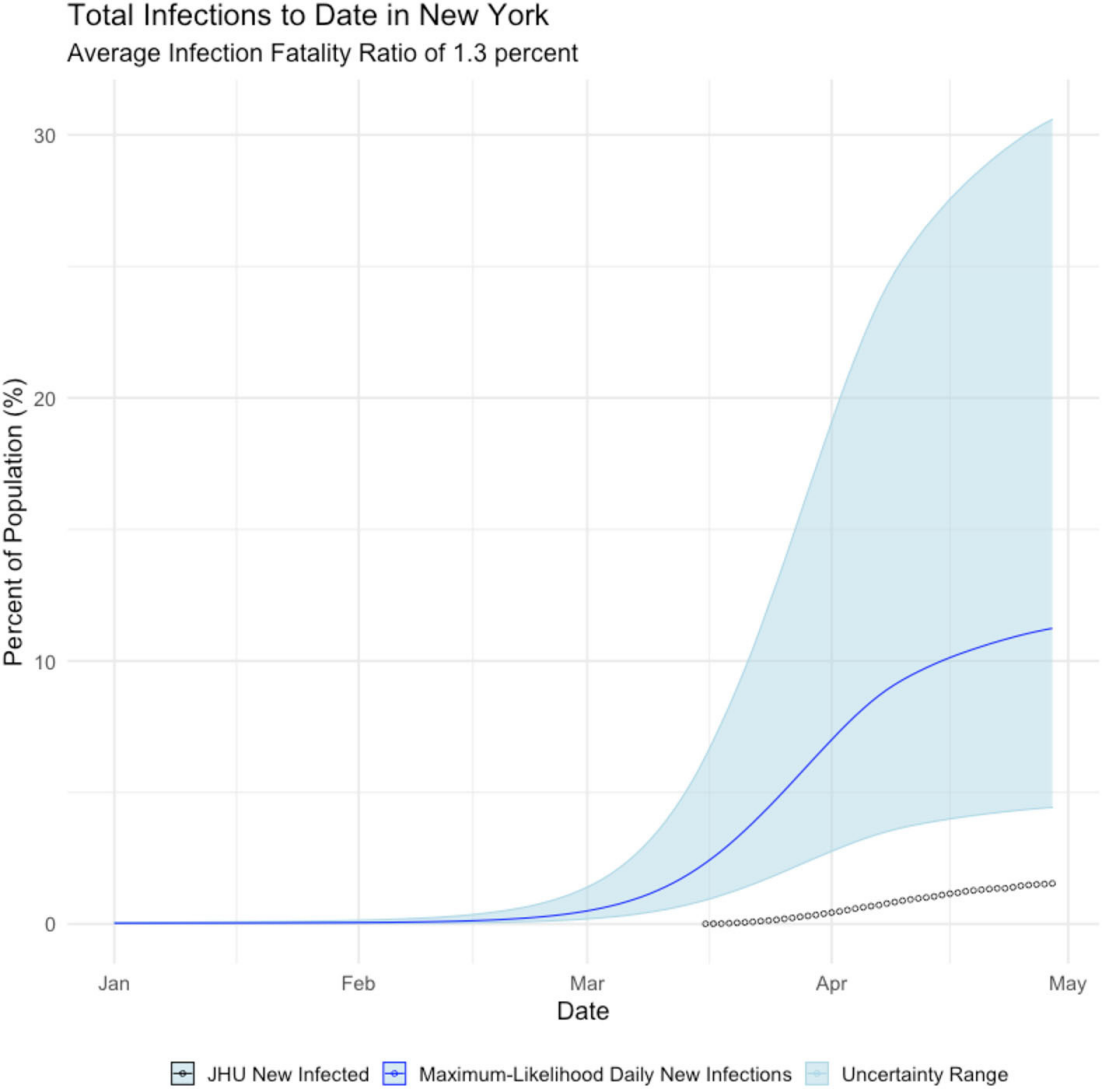
Daily Cumulative Infection count, New York state. Blue line is the maximum-likelihood estimate of daily new infections. Black circles are observed cases counts.

Figure 2, shows that New York state failed to detect the majority of *symptomatic* COVID cases until mid-April 2020—after the peak of the epidemic had passed. We further calculate that the true incidence of COVID in New York is cumulative infected population as of early May is over 2 million people, which is ~11% of New York residents, as shown below in Figure 3.

This finding can be validated with recent serology reports from New York. Serology reports from surveillance testing in New York City found that 12.3% of residents tested positive for COVID-19 antibodies as of early May, suggesting they had been infected with the virus (New York Press Office, 2020). The mean estimate of COVID19 cases is much closer to the extrapolated serology estimate than the reported confirmed positive case count of 1.8% of the population.

**Figure 4:**
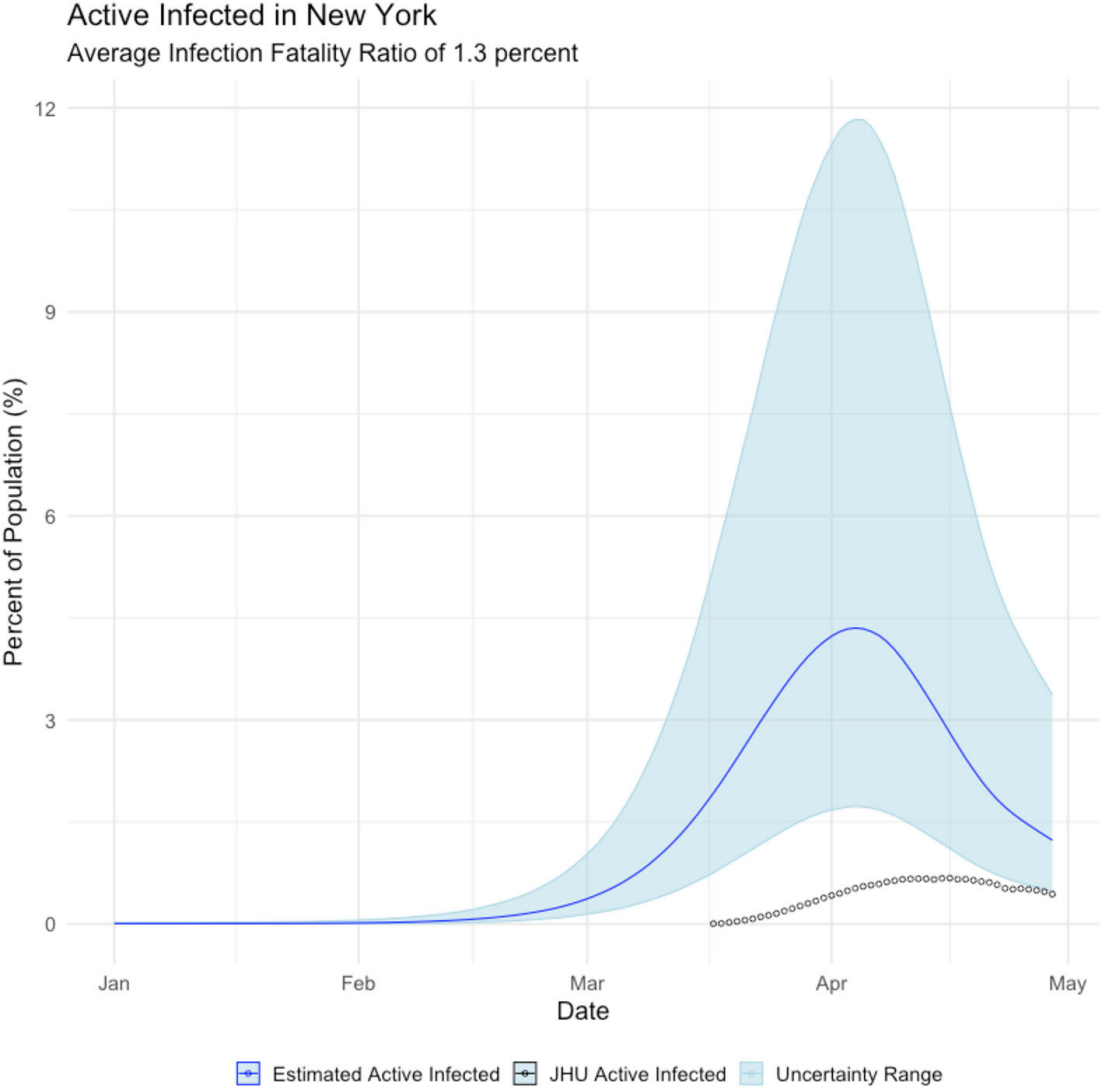
Relationship between estimated active infections (blue line), and active infections derived from the Johns Hopkins COVID19 site (black circles).

Undercounting those infected with the COVID19 virus affects the projection of when the disease will run its course and expire. Figure 4 displays the estimated mean daily infections using the proposed method with the daily infections derived from the Johns Hopkins University COVID19 site (Dong et al., 2020). It can be seen that the disease likely peaked much earlier than previously thought. Noting that while newly infected people peak in late March, most people report actively infected individuals, which peaks in early April. Regardless, the proposed method makes observation of the peak easier and the behavior of the epidemic clearer.

JHU case counts are reported as a cumulative data series. Thus, to observe the epidemic peak we must make assumptions to arrive at a prevalence curve. To calculate active cases *c*_*a*_(*t*) on day *t* we must make an approximation from the cumulative reported cases *c*_*r*_(*t*). We let γ represent the probability per day that an individual will recover from the virus. By inverting this metric we assert that it takes $\frac{1}{\gamma }$ days for an individual to recover from the virus and no longer be counted as an active case. To calculate *c*_*a*_(*t*) we sum over the new daily cases starting from $\frac{1}{\gamma }$ days into the past from the date for which we seek an active case count.

$$\begin{array}{l} c_{a}\left(t\right)=\overset{t}{\underset{i=t-\left(\frac{1}{\gamma }\right)}{\sum }}c_{r}\left(i+1\right)-c_{r}\left(i\right) \end{array}$$

Below we illustrate the mismatch between active cases as generated from our approach vs. what is being reported.

**Figure 5:**
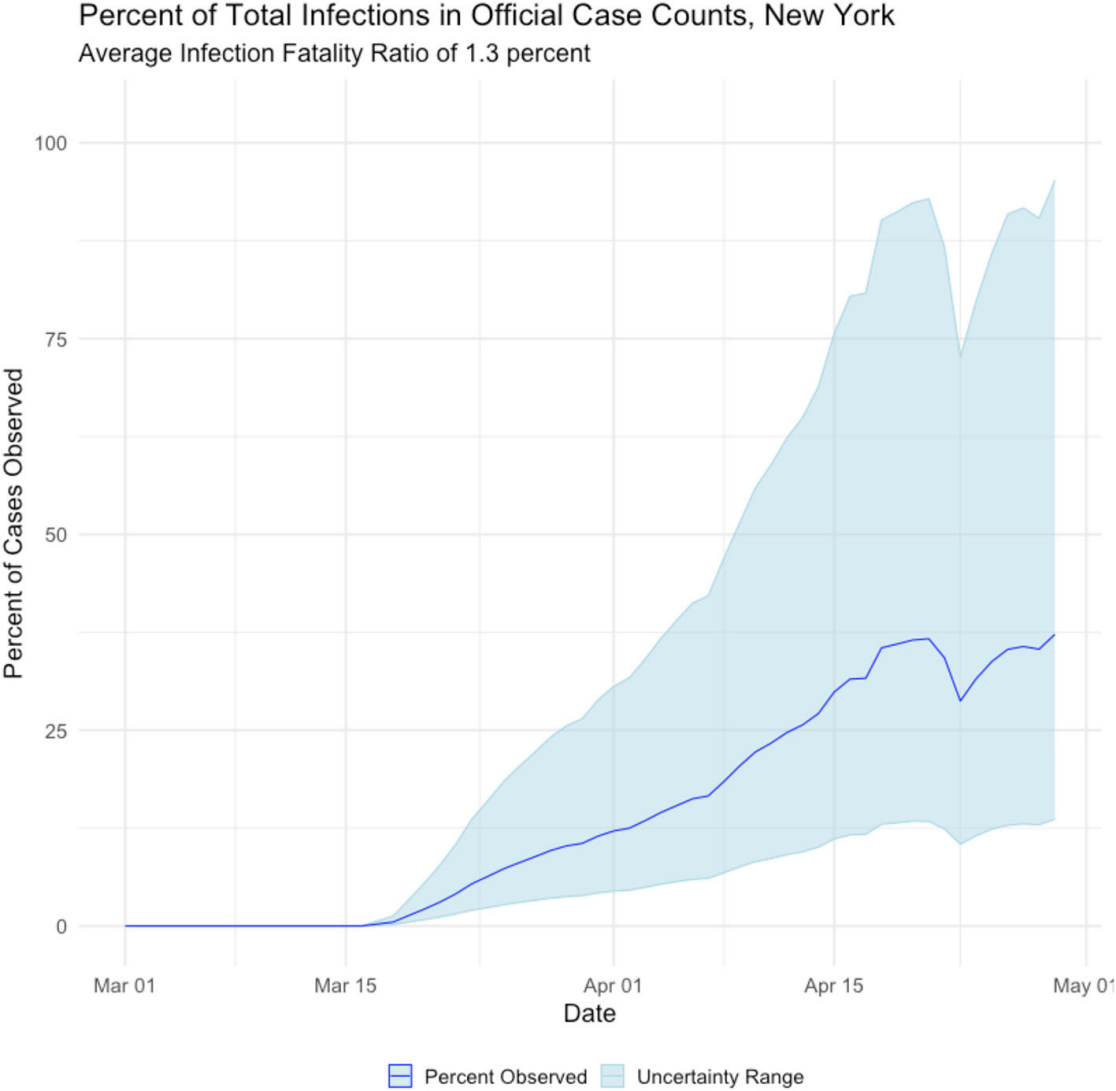
Observed Percentage of Estimates Reflected in Official Case Counts, New York state.

The proposed method of back estimating COVID19 cases from reported deaths provides additional insight into the accuracy of reporting. Figure 5 displays the percentage of estimated true cases that were reported in the Johns Hopkins COVID19 tracking site. It can be seen that the reporting improves significantly over time, which is expected given the media attention and public awareness the virus has received.

## International Examples

The above exercise was repeated for four notable international cases:

1. Mainland China, where the COVID outbreak originated and which observed a rapid dropoff in cases soon after the outbreak began;
2. Italy, which suffered the worst initial outbreak in the Western world (albeit soon superseded by the United States);
3. Germany, which seemingly suffered far fewer deaths than would be expected from its case totals; and,
4. Egypt, where the official infection rate appears disproportionately low for the population.

**Mainland China**


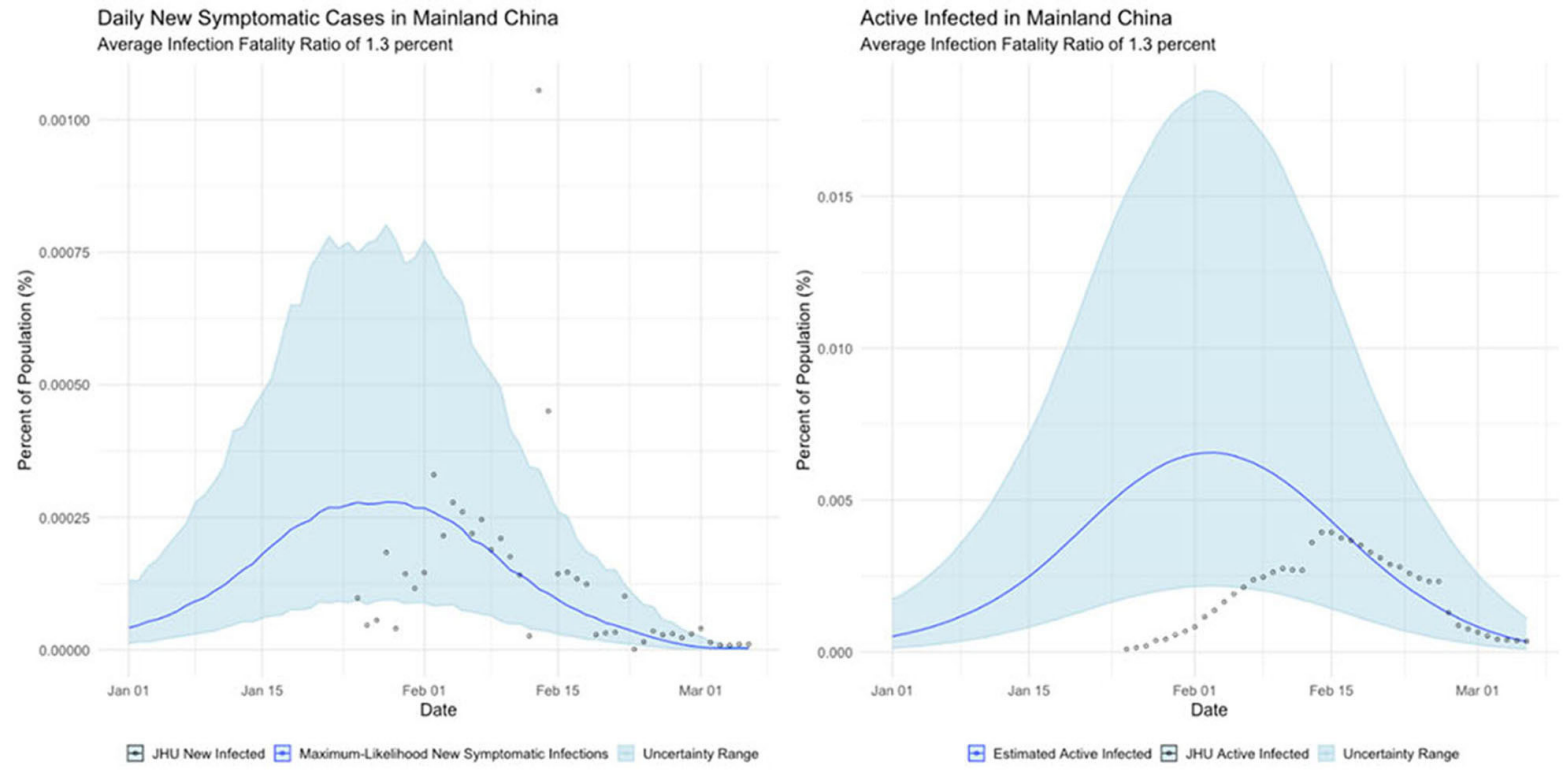


**Italy**


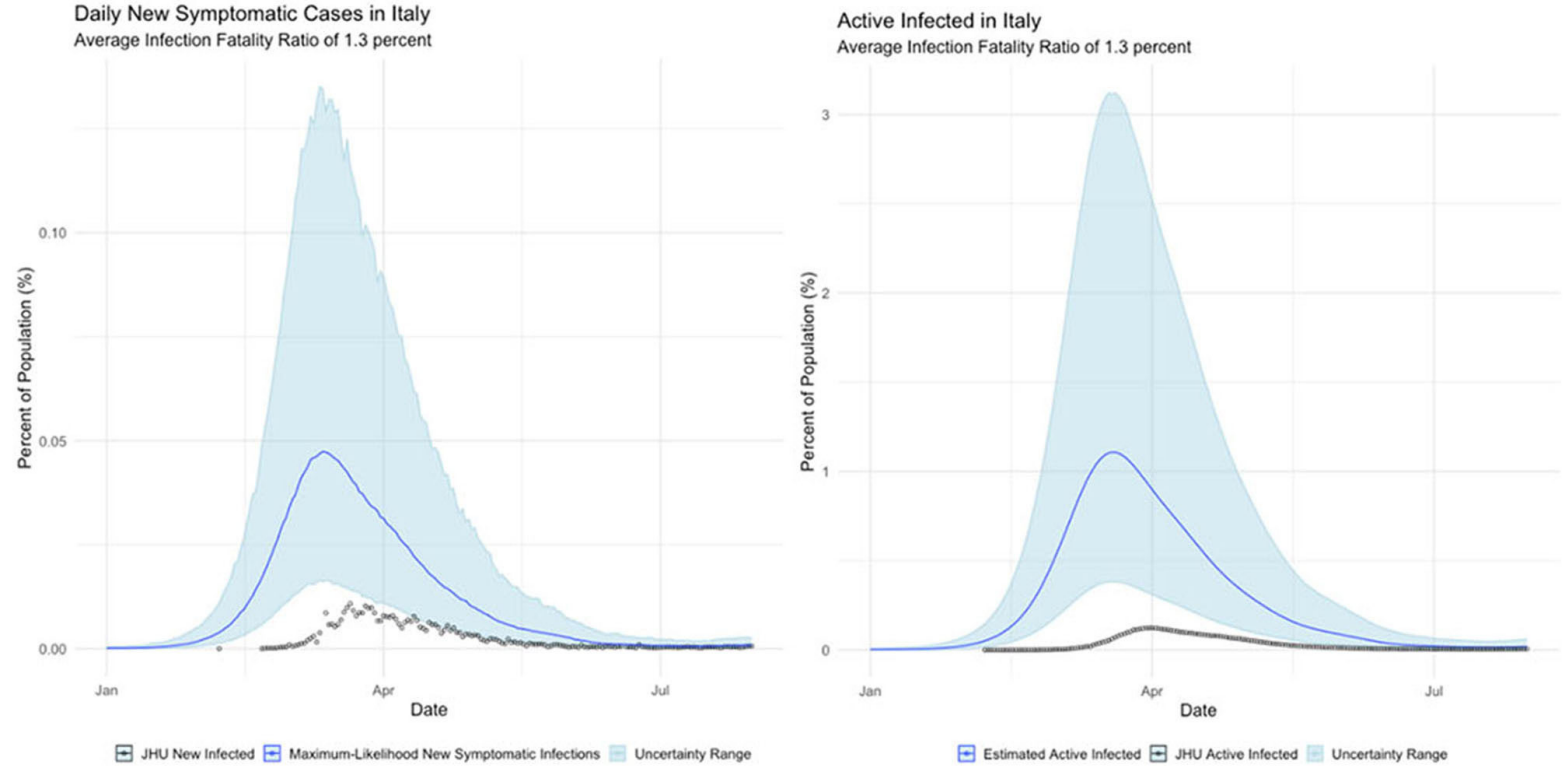


**Germany**


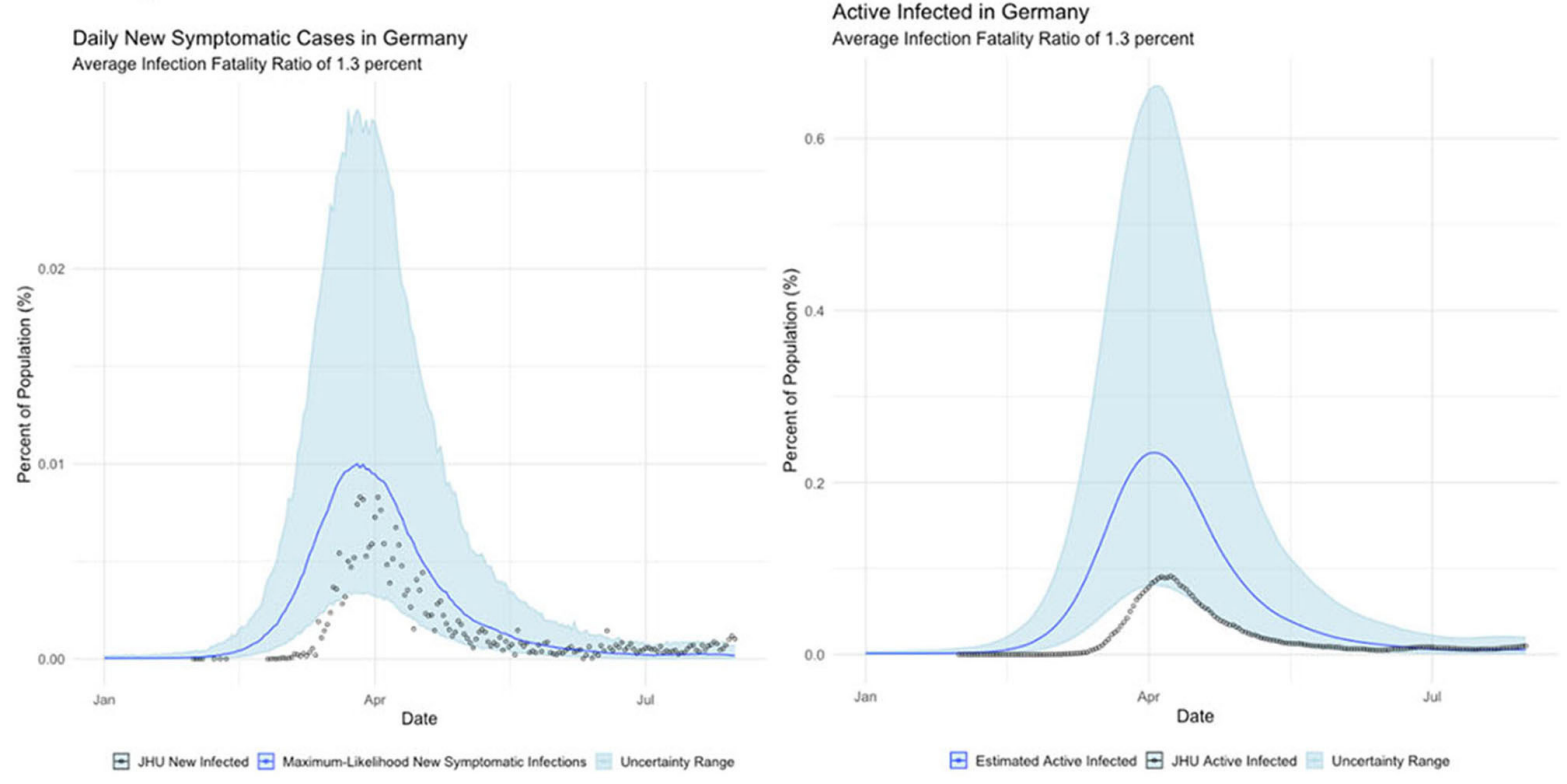


**Egypt**


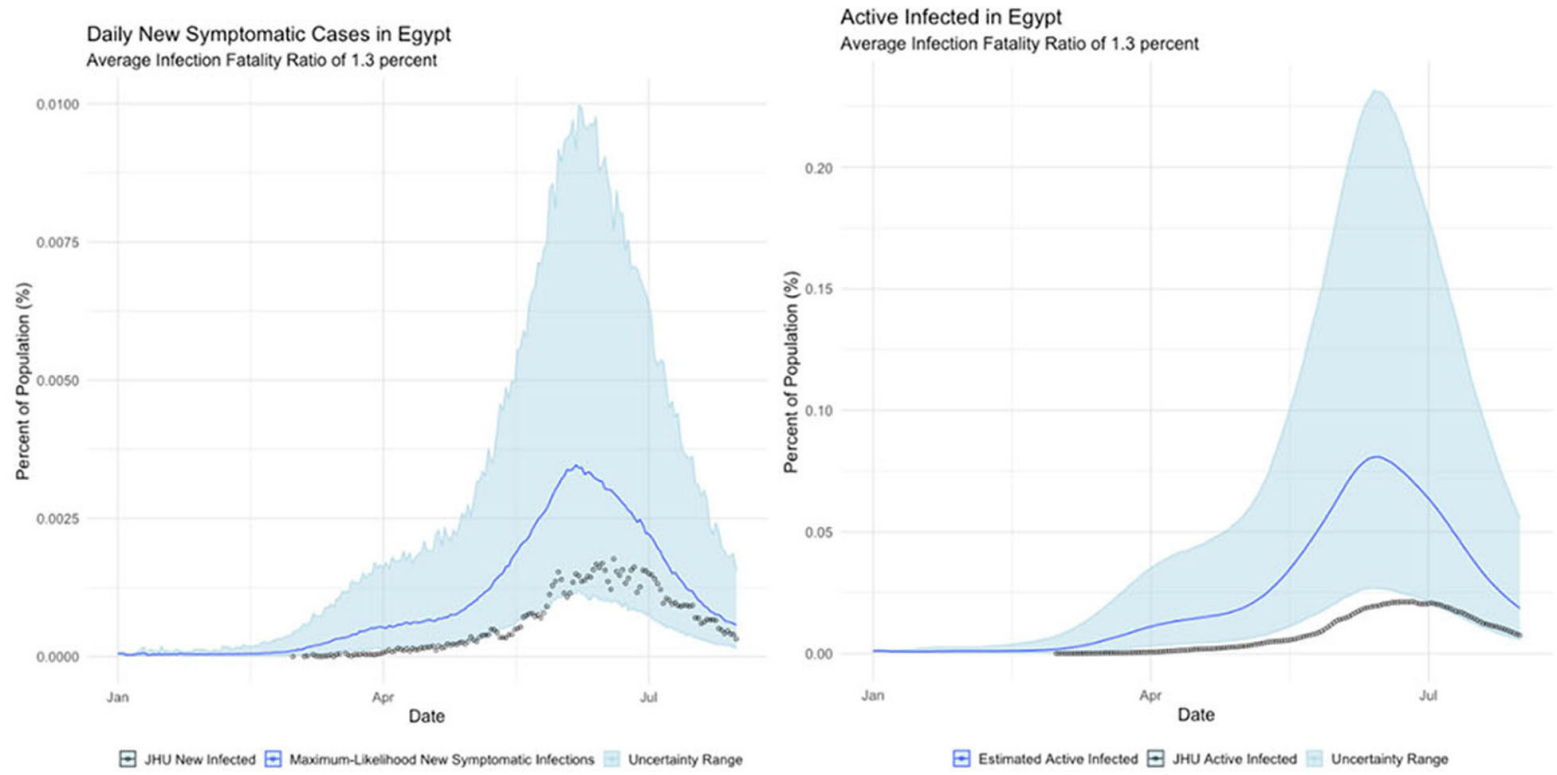


## Discussion

### New York

Three features stand out in the estimated data series. First, our analysis suggests over 2 million residents of New York state had been infected by the novel coronavirus as of 1 May, a far higher figure than estimates derived from confirmed case counts. Second, the observed peak of estimated infected people occurs in early April, several weeks earlier than case counts suggest. Third, the percentage of cases identified by confirmed case counts is a small and highly variable fraction of the total number of Infected.

New York state has a population of ~19.5 million people; therefore, our analysis suggests that between 4 and 29 percent of New York residents were infected by early May, with a most likely estimate of 11 percent. This estimate is validated by recent serology reporting from New York. Using the estimated COVID19 cases from the proposed method in epidemiology models, the behavior of the disease propagation is better explained, further suggesting estimation is preferred to confirmed case counts.

The observed poor agreement between confirmed case counts and our results was expected and is not especially troubling, in our view. Because COVID19 is a novel disease which has only recently been observed in humans, and because of widespread testing shortages, it is highly likely that the beginning of the outbreak in New York escaped detection altogether. It therefore makes intuitive sense that the observed percentage of cases would start at 0 and rise rapidly. Furthermore, unobserved early infections plausibly explain why our efforts to estimate for the reproduction rate *R*_***t***_, yielded non-sensically high estimates in the early phase of the outbreak.

### International Examples

The international examples considered in this paper reflect many of the same findings seen in New York state. Similar to New York, our estimates of COVID incidence in Mainland China, Italy, Germany, and Egypt indicate that the outbreaks in each country peaked before daily case counts would indicate. Additionally, the attack rate of the virus appears to be far higher in each example than could be derived from naïve case counts.

Additionally, our analysis of the international cases offers hints as to the resolutions to some of the seeming paradoxes found in their data. The Chinese data, for example, suggests that the epidemic had already peaked by the time case counts began to be formally recorded, which if true would neatly explain why Chinese cases dropped so precipitously.

Germany's COVID surveillance appears to have been the most rigorous of the countries we studied, as observed cases approach our maximum-likelihood estimate of symptomatic individuals. This conclusion is reinforced by the timing of the epidemic peak, which matches our estimates closely. The close agreement of observed and inferred case counts from Germany suggest that Germany is not actually uniquely successful at treating COVID patients, but rather that it detected the largest fraction of symptomatic cases.

Finally, Egypt and Italy appear to be more successful than the New York or China at detecting a representative fraction of true symptomatic COVID cases, but less successful than Germany.

## Conclusions

The implications of our findings are striking:

- First, it appears that lockdowns and/or individual decisions to socially distance produced a marked decline in new infections, as was the intended effect. New York does not appear to have achieved herd immunity to COVID19, although the large infected percentage may act as a headwind to further outbreaks there. Other states will likely benefit less from reduced population susceptibility, as New York had the United States' most severe outbreak to date.
- Second, the relationship between observed case counts and the true number of infections is so tenuous as to be useless for predicting or observing the trends in the true infection rate.
- Third, infections appear to have peaked earlier *in all cases* than case count data would imply. Furthermore, the level of agreement between the observed and inferred peaks in cases is an important clue as to the success of a state's disease surveillance regime—the closer the agreement, the more complete the surveillance of symptomatic cases.

Lastly, our approach has application beyond COVID19 and epidemic modeling. There are many big data problems that have issues with data quality and are similar to the COVID19 data problem. The data is drawn from and owned by many different, independent groups, with different data standards, platforms, motives, and restrictions. There are privacy and security considerations. Data ownership is not clear, and governance cannot be enforced. The proposed approach uses the most reliable data source available, findings from controlled or clinical studies, and well-understood mechanistic models to estimate the data from sources with poor quality. Identified differences can be used to direct cost-effective studies to validate modeled findings. In this manner, some data quality issues may be overcome and allow more effective modeling to inform situational awareness and decision support.

## Data Availability Statement

The raw data supporting the conclusions of this article will be made available by the authors, without undue reservation.

## Author Contributions

IM is the overall project lead directing the modeling effort and contributing author. KK is the primary author of this paper, proposed the methodology, and executed the code in TK assisted with modeling and is fitting the results to a follow-on SEIRD model. All authors contributed to the article and approved the submitted version.

## Conflict of Interest

All authors are employed by Accenture, a global consulting firm, which provides support to multiple agencies across the U.S. Federal Government. All work reported in this paper was conducted independently of any government or commercially funded work in an effort to advance academic, government and industry response to the global COVID19 pandemic.
